# Leptin stimulates migration and invasion and maintains cancer stem-like properties in ovarian cancer cells: an explanation for poor outcomes in obese women

**DOI:** 10.18632/oncotarget.4228

**Published:** 2015-05-22

**Authors:** Sumie Kato, Lorena Abarzua-Catalan, César Trigo, Ana Delpiano, Cristobal Sanhueza, Karen García, Carolina Ibañez, Katherine Hormazábal, Daniela Diaz, Jorge Brañes, Enrique Castellón, Erasmo Bravo, Gareth Owen, Mauricio Cuello

**Affiliations:** ^1^ Division of Obstetrics and Gynecology, Faculty of Medicine, Pontificia Universidad Católica de Chile, Santiago, Chile; ^2^ Department Hematology and Oncology, Pontificia Universidad Católica de Chile, Santiago, Chile; ^3^ Department of Physiological Sciences, Faculty of Biological Sciences, Pontificia Universidad Católica de Chile, Santiago, Chile; ^4^ Faculty of Medicine, University of Chile, Santiago, Chile; ^5^ Gynecologic Oncology Unit, Hospital Gustavo Fricke, Viña del Mar, Chile

**Keywords:** leptin, ovary, neoplasia, metastasis, obesity

## Abstract

The evidence linking obesity with ovarian cancer remains controversial. Leptin is expressed at higher levels in obese women and stimulates cell migration in other epithelial cancers. Here, we explored the clinical impact of overweight/obesity on patient prognosis and leptin's effects on the metastatic potential of ovarian cancer cells. We assessed clinical outcomes in 70 ovarian cancer patients (33 healthy weight and 37 overweight) that were validated with an external cohort from The Cancer Genome Atlas (TCGA) database. Progression-free and overall survival rates were significantly decreased in overweight patients. Similarly, a worse overall survival rate was found in TCGA patients expressing higher leptin/OB-Rb levels. We explored serum and ascites leptin levels and OB-Rb expression in our cohort. Serum and ascites leptin levels were higher in overweight patients experiencing worse survival. OB-Rb was more highly expressed in ascites and metastases than in primary tumors. Leptin exposure increased cancer cell migration/invasion through leptin-mediated activation of JAK/STAT3, PI3/AKT and RhoA/ROCK and promoted new lamellipodial, stress-fiber and focal adhesion formation. Leptin also contributed to the maintenance of stemness and the mesenchymal phenotype in ovarian cancer cells. Our findings demonstrate that leptin stimulated ovarian cancer cell migration and invasion, offering a potential explanation for the poor prognosis among obese women.

## INTRODUCTION

Epithelial ovarian cancer is the deadliest gynecologic malignancy [1]. Despite extensive efforts to improve the detection and treatment of ovarian cancer, the majority of affected women still succumb to the disease. A number of factors contribute to this outcome, including a late diagnosis, the absence of highly curative chemotherapy and the high degree of molecular heterogeneity among ovarian cancers [2]. In total, the overall cure rate for newly diagnosed ovarian cancer in women is less than 40% across all stages [3].

Several groups have attempted to identify modifiable factors contributing to ovarian carcinogenesis that can be targeted for new therapeutic strategies. Potential modifiable risk factors that have already been explored include diet, exercise, cigarette smoking, alcohol intake and obesity. For most of these factors, the evidence is conflicting or inconsistent and does not support an association with ovarian cancer risk [2]. However, accumulating evidence has begun to support the role of obesity in ovarian carcinogenesis. In this respect, two epidemiological studies have established an association between ovarian cancer and body mass index (BMI), a strong marker for obesity [4, 5]. Consistent with these findings, our group has shown a 20% increase in the ovarian cancer age-adjusted mortality rate among Chilean women, a population where the obesity prevalence has strikingly risen above 30% in recent decades [6]. This increase in mortality is not related to changes in life expectancy or difficulties in accessing adequate treatment in a developing country.

How obesity contributes to ovarian carcinogenesis and influences its behavior is not fully understood. For ovarian cancer, one potential explanation that has been proposed is the ‘inflammation theory’. This hypothesis contends that chronic exposure of the ovarian epithelium to inflammatory stimuli triggers malignant transformation and subsequently favors a more aggressive behavior of cancer cells [7]. Accumulated adipose tissue deposits and a state of chronic low-grade inflammation characterize obesity. In this activated state, adipocytes and inflammatory cells secrete adipokines and several inflammatory cytokines that have been associated with tumor progression and metastasis in other epithelial cancers, including breast, colon and prostate cancer [8].

Leptin and adiponectin are two adipokines that are abnormally secreted in obese women. Typically, serum leptin levels are elevated in obese patients, while adiponectin levels are decreased [9]. Leptin is a peptide hormone that plays a major role in regulating the energy intake and expenditure processes, including appetite and metabolism. Leptin acts through its receptor (Ob-R), which is encoded by the *LEPR* gene [10]. OB-Rb is the predominant, fully functional isoform that is responsible for the biological actions of leptin [11]. This isoform has been identified in several epithelial cancers, including thyroid cancer, hepatocellular carcinoma, breast cancer and colon cancer [12]. Upon leptin binding to OB-Rb, there is concomitant activation of the JAK/STAT, MAPK and PI3K/AKT signaling pathways, leading to cell proliferation and migration. [13–17]. Recent studies have suggested that higher circulating levels of leptin, higher leptin receptor expression by the tumor and a high leptin to adiponectin (L:A) ratio all correlate with a worse outcome in several epithelial cancers, including ovarian cancer [18, 19]. Little is known regarding leptin's effects on ovarian cancer cells. *In vitro* studies performed in BG-1, SKOV3 and OVCAR-3 cancer cells have shown that leptin stimulates cell growth and inhibits apoptosis [14, 20]. No findings have been reported regarding leptin's effects on the migration and invasion of ovarian cancer cells or the dominant signaling pathways.

Cell migration is a crucial multistep process in many chronic inflammatory diseases, including cancer [21, 22]. Migration involves changes in the actin cytoskeleton and the formation and turnover of protein complexes within focal adhesions and in the extracellular matrix [23, 24]. The key molecules regulating this process are the Rho family of GTPases. Several chemokines and growth factors released within the tumor microenvironment act as driving forces in this process by regulating Rho activity (e.g., IL-6, EGF) [21]. To migrate and invade, epithelial cancer cells must undergo the epithelial-mesenchymal transition (EMT). Activation of the EMT program confers not only the ability to metastasize into cancer cells but also the property of self-renewal that is crucial for clonal expansion at the dissemination site [25]. In most cancers, it is possible to isolate a small subset of cancer cells that express EMT and stemness markers; this subset, termed cancer-initiating cells (CICs), adapt and respond to environmental stimuli (e.g., IL-6, EGF) to invade and metastasize [25, 26]. The leptin receptor shares structural homology with other cytokine family members, including IL-6, which is known to be involved in the EMT of ovarian cancer cells. Therefore, it is reasonable to hypothesize that leptin can also act as a regulator of the metastatic process [10, 26].

Based on these facts, we postulated that the leptin/OB-Rb pathway could contribute to ovarian cancer recurrence and progression, particularly in obese women, resulting in a worse survival rate.

## RESULTS

### An overweight status is associated with worse progression-free and overall survival in platinum-sensitive epithelial ovarian cancer

**Figure 1:**
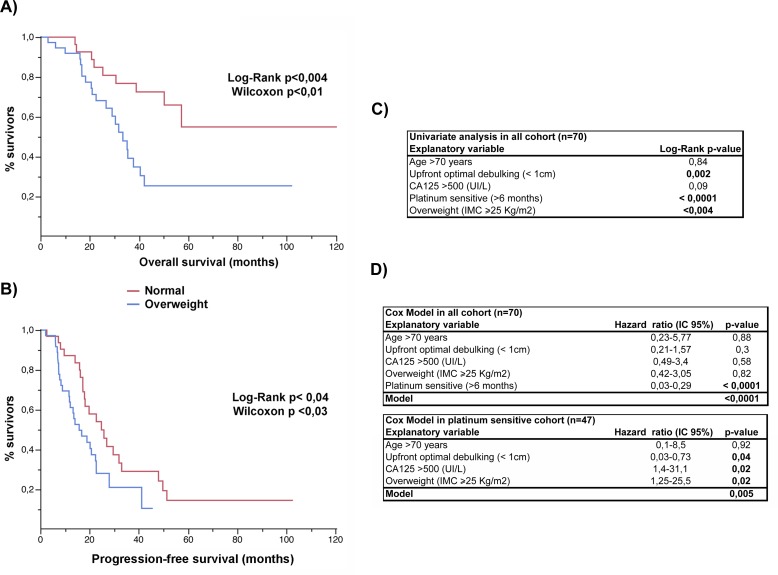
Overweight status is an independent factor affecting outcome in ovarian cancer Comparison of the **A.** overall and **B.** progression-free survival curves between the healthy BMI (normal) and overweight subgroups. **C.** Univariate analysis and **D.** the Cox model of potential explanatory variables affecting the overall survival in epithelial ovarian cancer.

To address whether obesity constitutes a risk factor that predisposes a worse outcome in epithelial ovarian cancer, we studied 70 stage III and IV patients that were treated at our institution and stratified the cases by BMI (healthy weight, BMI < 25 kg/m^2^; overweight, ≥25 kg/m^2^). The clinical demographics of the study cohort are summarized in Table 1. The average BMI was 22.1±2 Kg/m^2^ and 28.9±4 Kg/m^2^ in the healthy and overweight groups, respectively (*t*-test, *p* < 0.0001). The overweight group was significantly older than healthy BMI group (*t*-test, *p* = 0.02). There were no significant differences in histology or stage distribution, CA125 levels at diagnosis, the percentage of primary optimal debulking ( < 1 cm), neoadjuvant therapy, sensitivity to the platinum-based scheme, access to second or third line or secondary cytoreduction between groups. As shown in Figure 1, four variables were identified as negative factors in terms of progression-free and overall survival for the cohort. These factors included achievement of optimal debulking at primary surgery, CA125 > 500 UI/L, sensitivity to platinum-based chemotherapy ( > 6 months), and overweight status (survival curves and univariate analysis are shown in Figure 1A, 1B and 1C). Additionally, we performed a deeper analysis by stratifying BMI into 4 categories (< 18.5, ≥18.5− <25, ≥25− < 30, ≥30 Kg/m^2^) or using a continuous variable. In both scenarios, this variable remained as a significant negative factor in the survival analysis (see Supplementary Figure 1). The strongest effect for a negative factor was sensitivity to chemotherapy. Its inclusion in the Cox model abolished the effects of the other variables in the cohort (upper table in Figure 1D). However, a subgroup analysis restricted to platinum-sensitive patients demonstrated that the other factors, including overweight/obesity, also had independent effects on survival (lower table in Figure 1D).

**Table 1:** Clinical demographics of the study population

Variable		Normal	Overweight	p-value
**n (%)**		**33 (47,1)**	**37 (52,9)**	
**Weight (kgs)**		**55,9 ± 1,6**	**71,2 ± 31,5**	0,0001
**IMC (Kg/mt2)**		**22,1 ± 2**	**28,9 ± 4,4**	0,0001
**Age (years)**		53,2 ± 9,5	58,6 ± 8,8	0,02
**Stage**	**III**	28 (87.5)	33 (89.2)	NS
	**IV**	4 (12,5)	4 (10,2)	
**Histology**				
**Serous-papillary: n (%)**		**24 (72,7)**	**30 (81,1)**	NS
**High grade (G3)**		21 (87.5)	29 (96.7)	NS
Mucinous: n (%)		0 (0)	1 (2,7)	
Other: n (%)		9 (27,3)	6 (16,2)	
**CAl25 (UI/L)**		1953,5	2439,9	NS
**Primary cytoreduction**	**no**	11 (36.4)	18 (48.6)	
	**yes**	19 (57.6)	16 (43,2)	NS
	**intent**	3 (9,1)	3 (8,1)	
**Upfront optimal debulking (<1 cm) n (%)**		**19 (57,6)**	**15 (40,5)**	NS
**Neoadyuvant therapy: n (%)**		**9 (27,3)**	**19 (51,4)**	0.05
**Interval cytoreduction: n (%)**		8/9 (88.9)	14/19 (73.7)	NS
**First line chemotherapy**				
carboplatin-paclitaxel: n (%)		24 (72,7)	28 (75,7)	NS
cisplatin-cyclophosphamide: n (%)		2 (6,1)	3 (8.1)	
other: n (%)		7 (21.2)	6 (16,2)	
**n° cycles: n (%)**	**6**	24 (72.7)	29 (78.4)	NS
	**<6**	5 (15,2)	5 (13,5)	
	**>6**	4(12,1)	3(8.1)	
**Optimal debulking in interval surgery**		7/8 (87.5)	10/14 (714)	NS
**Total optimal debulking upfront/interval**		**26/33 (78,8)**	**25/37 (67,6)**	NS
**Nadir Ca125 after last chemo cycle**		179,8 ± 124,2	101,9 ± 116,7	NS
**Resistant to platinum (<6 m)**		**6 (18,2)**	**11 (29,7)**	NS
**Recurrent disease**		21 (63.6)	27 (73)	NS
**Secondary cytoreduction**		5/21 (23,8)	3/27 (11,1)	NS
**Second line chemotherapy: n (%)**		18/21 (85,2)	18/27 (66,7)	NS
**Third line chemotherapy: (%)**		8/21 (38,1)	11/27 (40,7)	NS

A summary of the clinical demographics comparing healthy BMI (normal: ≥18.5 to <25 kg/m^2^) versus overweight (≥25 kg/m^2^) patients.

### Differential OB-Rb expression is found in type I and II ovarian cancers, and higher circulating leptin levels are present in the overweight subgroup

**Figure 2:**
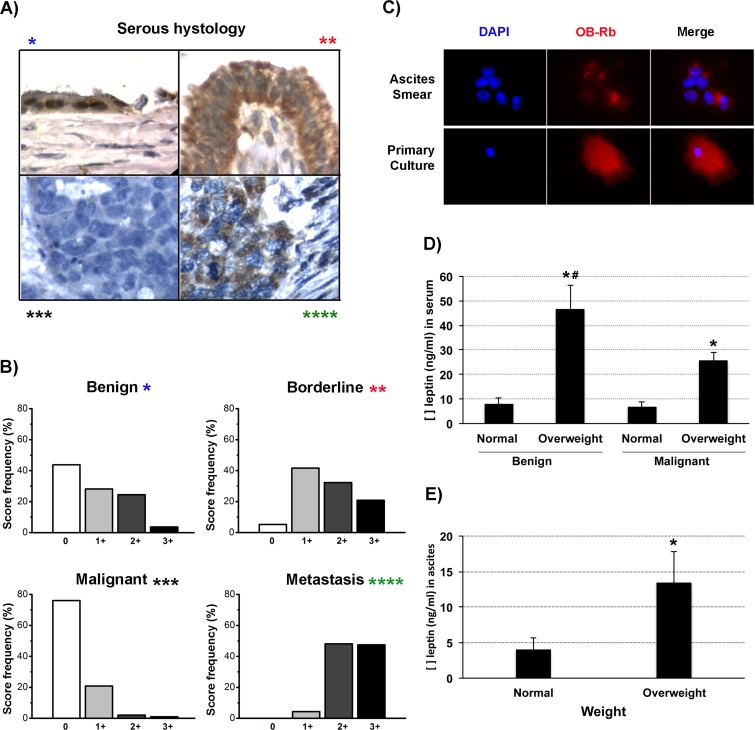
OB-Rb and leptin expression levels in epithelial ovarian cancers **A.** Measurement of OB-Rb expression in serous ovarian samples by immunohistochemistry (benign *, borderline **, malignant primary tumor ***, and metastatic lesion ****). **B.** The relative frequency (%) of +, ++, +++, and ++++ OB-Rb expression levels was digitally scored in 10 benign, borderline, malignant and metastatic lesions. **C.** OB-Rb expression was measured by immunofluorescence in an ascites smear and a primary tissue culture established from the ascites collected from a patient with stage IIIC serous papillary ovarian cancer. **D.** ELISA measurement of the circulating leptin levels (ng/ml) in healthy BMI (normal) and overweight patients undergoing surgery for benign or malignant conditions. **E.** ELISA measurement of the ascites leptin levels in healthy BMI (normal) and overweight ovarian cancer patients. * Indicates a significant difference (*p* < 0.05) between the leptin levels measured in overweight and healthy BMI patients. # Indicates a significant difference (*t*-test, *p* < 0.05) between the benign and malignant overweight patients. Bars in the columns indicate the standard deviation.

High OB-Rb expression and high circulating levels of leptin have been correlated with poorer outcomes in epithelial ovarian cancer [18]. Therefore, we first evaluated OB-Rb expression levels in primary tumors, ascites and metastatic tumors and identified any correlation with disease progression. Samples were collected from patients affected by type I (serous borderline and low-grade tumors) and type II (serous high-grade tumors) ovarian cancers. We also collected samples from benign serous lesions for comparison. Most samples corresponded to patients suffering from overweight or obesity. Using a digital score (+ to ++++), we identified a tendency towards increased intensity of OB-Rb expression in type I ovarian cancers compared with the benign lesions (Figure 2A, * represents benign lesions, ** represents borderline or type I cancer). We also observed an inverse correlation between OB-Rb expression and the histological grade in the primary tumors (Figure 2A, *** represents malignant tumors). Lower levels of OB-Rb expression were observed in high-grade serous ovarian cancers (Figure 2A, lower left picture in serous panel and Figure 2B). Interestingly, OB-Rb expression tended to increase again in the ascites (Figure 2C) and metastatic lesions (either at mesothelium or omentum) in high-grade serous lesions, with a score pattern similar to that observed in type I ovarian cancers (lower right images in the serous panel in Figure 2A and Figure 2B).

Then, we measured the circulating leptin levels in healthy and overweight patients undergoing surgery for benign and malignant lesions. Leptin levels were significantly higher in the overweight patients compared with the healthy BMI patients. Despite the fact that the leptin levels were significantly lower in the cancer patients compared with the benign-overweight group, the levels were still significantly higher than the benign or malignant healthy BMI counterparts (Figure 2D). Because circulating leptin levels do not necessarily reflect the ascites leptin levels, we also measured leptin levels in ascites samples collected from overweight and healthy BMI cancer patients. As shown in Figure 2E, ascites leptin levels were also significantly higher in the overweight patients.

### Primary tissue culture and ovarian cancer cell lines express the active form of the leptin receptor

As mentioned above, higher OB-Rb and leptin expression levels were found in ascites and metastatic tumors, particularly in overweight patients. These higher levels also correlated with a worse overall survival. Therefore, we explored the role of this pathway in key steps controlling the metastatic potential, which are cellular EMT, migration and invasion.

**Figure 3:**
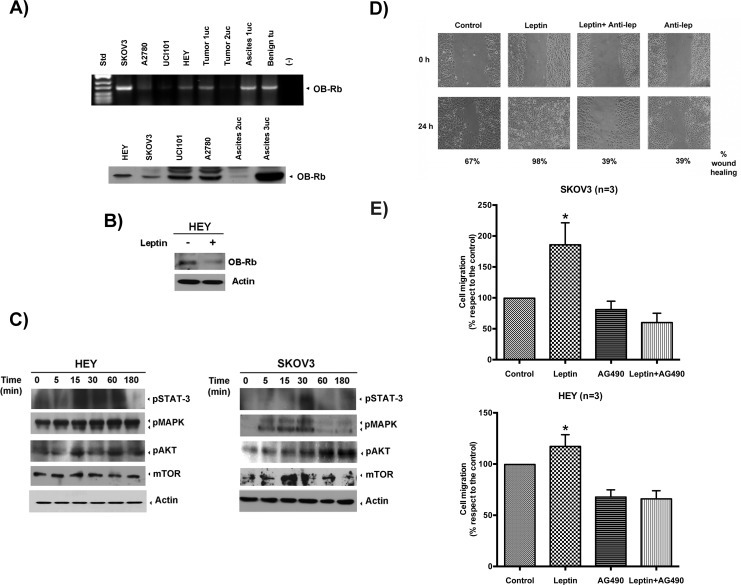
OB-Rb expression in ovarian cancer cell lines and primary tissue cultures and leptin's effects on cell proliferation, cell migration and invasion **A.** OB-Rb expression in benign and malignant epithelial ovarian cell lines was measured by RT-PCR (upper panel) and immunoblot analyses (lower panel). **B.** The biotinylated OB-R receptor was measured by immunoblotting in HEY cells treated with vehicle or leptin (100 ng/ml for 30 min). **C.** The phosphorylated forms of the downstream kinases (STAT-3, MAPK, AKT, mTOR) were determined by immunoblotting during a time course of leptin exposure. Actin is shown as a loading control. **D.** Representative experiments of the effects of a leptin-neutralizing antibody on leptin-induced cell migration in SKOV3 cells. **E.** Effect of the JAK inhibitor, AG490, on leptin-induced (100 ng/ml, 12 or 24 h) cell migration (*upper panel*) and invasion (*lower panel*) in SKOV3 and HEY cells as measured by wound-healing and Boyden chamber assays, respectively. The data represent the mean±SD of at least three different experiments. # Indicates a significant difference between leptin and control. * Indicates a significant difference between leptin and leptin plus AG490.

We first examined whether the human ovarian cancer cell lines and the primary tissue cultures expressed the active isoform of the leptin receptor, OB-Rb. As shown in the upper panel of Figure 3A, the ovarian cancer cell lines (SKOV3, HEY, UCI101, A2780 and OVCAR3), the primary tissues cultures established from patients with stage III serous-papillary epithelial ovarian cancer (named tumor 1 with associated ascites 1 and tumor 2) and the primary tissue culture established from a benign ovarian tumor all expressed the OB-Rb isoform, as measured by conventional RT-PCR. As shown in the lower panel of Figure 3A, all cancer cell lines and the two primary tissue cultures established from the cancer patients (named ascites 2 and 3) expressed the OB-Rb isoform when analyzed by immunoblotting.

For further experiments, we chose to investigate leptin's effects in two ovarian cancer cell lines: the p53-wild type, BRAF-mutated HEY cells and the p53-null SKOV3 cancer cells. Additionally, we confirmed these effects in primary tissue cultures established from patients affected by advanced-stage epithelial ovarian cancer. The rationale was to work with cell lines that match the two major molecular profiles (type I and II serous ovarian cancers) described for epithelial ovarian cancer [27] and that express different OB-Rb levels.

To confirm that the receptor isoform was active, we exposed the cells to multiple leptin concentrations for varying lengths of time. After treatment, we explored receptor internalization, the downstream signaling pathways known to be activated by leptin binding in other epithelial cancers (e.g., JAK/STAT3, MAPK, AKT and mTOR) and the effects on cell growth, migration and invasion. Upon ligand binding, OB-Rb is internalized (Figure 3B) in these cancer cells, and the downstream signaling cascades are also activated (Figure 3C). Leptin stimulation also resulted in an increase in cell migration and invasion in both cell lines (Figure 3D and 3E). A similar effect on cell invasion was observed in one primary tissue culture established from a stage IIIC, high-grade serous ovarian cancer (see Supplementary Figure 3A).

To confirm that the increase in cell migration and invasion was mediated by the activation of the leptin receptor and the downstream signaling pathway mentioned above, we pre-incubated SKOV3 cells with an anti-OB-R blocking peptide and selective inhibitors for the kinases that act at the early and late steps of this pathway. As shown in Figure 3D, pre-incubation with the anti-OB-Rb-neutralizing antibody almost completely abrogated leptin-induced migration in SKOV3 cells, confirming that this effect requires OB-Rb activation. In addition, pre-incubation with AG490, an inhibitor of JAK, the first kinase in the leptin activation cascade, significantly reduced the phosphorylation of other downstream target kinases (e.g., MAPK, shown in Supplementary Figure 3B). It also inhibited leptin-induced cell migration and invasion in these cells (Figure 3E upper and lower panels, respectively). A similar effect was also observed for cell migration using LY294002, an inhibitor affecting the PI3K/AKT kinases located downstream of JAK in the leptin cascade (Supplementary Figure 3C).

It was previously shown that leptin induces cell growth in ovarian cancer cells [14]. Therefore, we decided to reaffirm this finding in our cancer cells and performed cell proliferation assays using a wide range of concentrations (0-1000 ng/ml) and exposure times (0-72 h). In contrast to the findings reported by others, leptin induced only a slight and non-significant increase in cell proliferation in two of the four cell lines we tested (A2780 and UCI101 cells; upper panel Supplementary Figure 3D), despite using different leptin doses. Only longer incubations (48-72 h; lower panel Supplementary Figure 3D) resulted in an increase in cell growth similar to that described in a previous report [14].

### Leptin induces the formation of new focal adhesion complexes and stress fibers in ovarian cancer cells by activating RhoA

**Figure 4:**
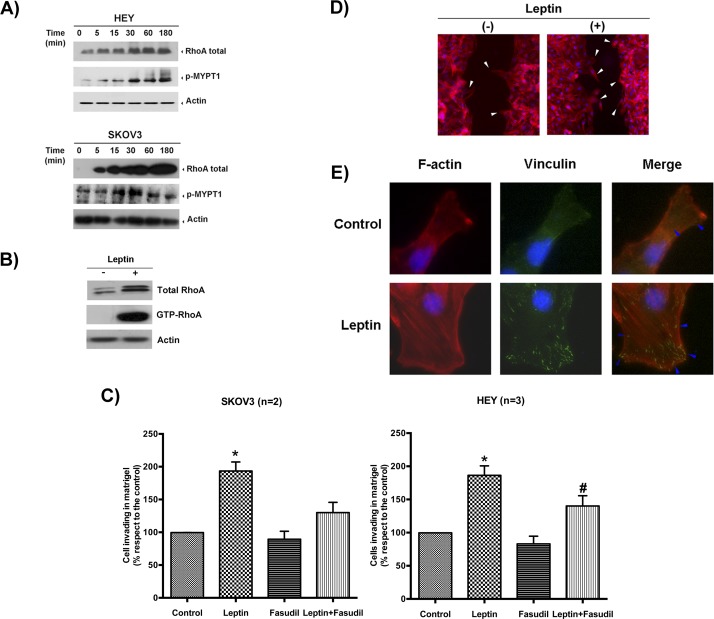
Leptin stimulates cell migration and invasion by activating the RhoA/ROCK pathway **A.** Leptin effects (100 ng/ml, time course) on RhoA protein levels and MYPT1 phosphorylation in HEY and SKOV3 cells were measured by immunoblotting. Actin was used as a loading control. **B.** Total RhoA and GTP-RhoA (upon pull-down assay) were measured by immunoblotting in HEY cells treated with mock or leptin (100 ng/ml for 30 min). Actin is shown as a loading control. **C.** Effects of fasudil in leptin-induced cell invasion in both cell lines (leptin: 100 ng/ml, 24 h). * Indicates a significant difference between leptin and the other conditions. # Indicates a significant difference between the leptin-treated cancer cells in the absence or presence of the inhibitor. **D.** Detection of lamellipodial formation in the edges of the wound by F-actin immunocytochemistry in HEY cells treated with vehicle or leptin (100 ng/ml for 30 min). **E.** Detection of new focal adhesion formation in HEY cells treated with the same conditions described above using F-actin and vinculin immunocytochemistry and confocal microscopy.

Rho GTPase family members are key players in the critical steps of cell movement [21]. Several of its members are regulated by leptin-triggered kinases (e.g., MAPK, AKT). As shown in Figure 4A, a significant increase in the total RhoA protein levels (member of Rho family) was observed shortly after leptin incubation, but was nearly undetectable under basal conditions. We also observed an increase in the phosphorylation of the MYPT1 isoform, a phosphatase that regulates cytoskeletal reorganization in response to RhoA/ROCK signaling, starting between 5 to 15 min after treatment. The increase in the total RhoA levels and MYPT1 phosphorylation suggests that the ROCK/RhoA signaling pathway was activated. To assess whether this increase in total RhoA corresponded to an increase in the active form, we performed a RhoA pull-down assay. As shown in the immunoblot in Figure 4B, a 30-min incubation with leptin increased levels of both the total RhoA protein as well as its active form, GTP-RhoA, in HEY cells compared with the control. To reaffirm that the ROCK/RhoA signaling pathway regulated leptin-induced cell migration and invasion, we added fasudil hydrochloride, a selective inhibitor of RhoA/ROCK. As shown in Figure 4C, fasudil pre-incubation completely abrogated cell invasion and migration in both cell lines (see also Supplementary Figure 4).

The critical steps for cell migration are the formation of new focal adhesion complexes and cell body contraction (stress-fiber assembly and contraction) [28]. To demonstrate that leptin was prompting these steps, we first performed a wound-healing assay and immunofluorescence detection of leptin-stimulated lamellipodial formation in HEY cells. Six hours after the wound was created, the cells were fixed and stained for F-actin with rhodamine-labeled phalloidin. As observed in Figure 4D, the leptin-treated cells exhibited many lamellipodial protrusions at the wound edges, as shown by an increase in F-actin staining relative to the control. To confirm that leptin was indeed stimulating the formation of new focal adhesion complexes and stress-fiber assembly, we repeated the previous experiment under similar conditions, but added fluorescein-labeled vinculin. Vinculin is a membrane-cytoskeletal protein in focal adhesion plaques that is involved in linking integrin adhesion molecules to the actin cytoskeleton. As shown in Figure 4E, leptin induced stress-fiber assembly (F-actin panel) and the formation of new focal-adhesion complexes in the leading edge of the wound (vinculin panel).

### Leptin induces EMT and stemness-related gene expression and the invasion of ovarian cancer-initiating cells

**Figure 5:**
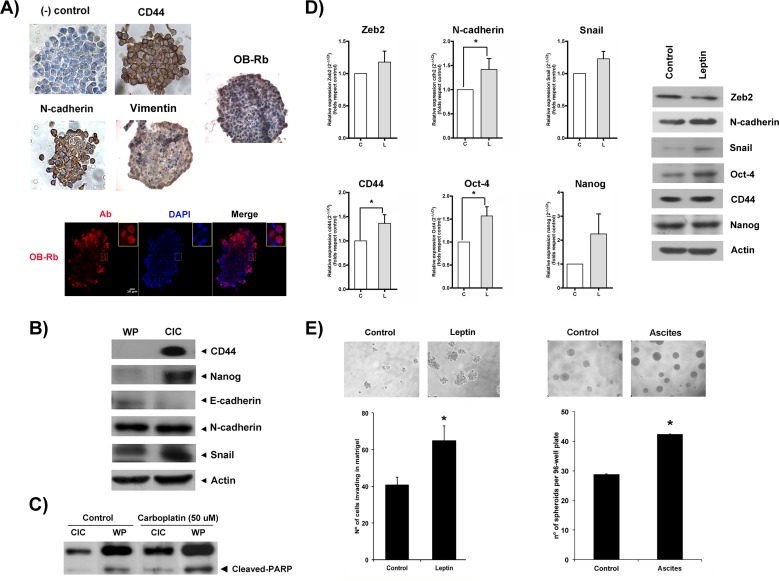
Leptin contributes to the maintenance of stemness and EMT expression in ovarian cancer cell subpopulations and prompts spheroid formation and cancer cell invasion **A.** OB-Rb expression, stemness (CD44), and EMT markers (N-cadherin, Vimentin) were characterized in HEY cancer-initiating cells (CIC) by immunocytochemistry and immunofluorescence (DAPI: nuclear staining, Ab: antibody). **B.** The comparative expression of stemness (CD44, Nanog) and EMT markers (N-cadherin, E-cadherin, and Snail) were measured in CICs and the whole population of HEY cells (WP) by immunoblot analysis. **C.** Cleaved PARP was detected in vehicle- or carboplatin-treated (50 μM for 24 h) CICs and the WP of HEY cells by immunoblot analysis. **D.** The leptin-induced effects on the RNA (*left panels*) and protein (*right panels*) expression of stemness (CD44, Oct-4, and Nanog) and EMT markers (cdh2 or N-cadherin, Zeb2, Snail) were measured in CICs treated with vehicle or leptin (100 ng/ml for 6 h) by real-time PCR and immunoblotting. **E.** HEY spheroid formation (*upper and right lower panels*) and CIC invasion (*left lower panel*) were assessed in Matrigel Boyden chamber assays with vehicle, leptin or ascites treatment. The bar graph summarizes the mean±SD of three Matrigel invasion assays. * Indicates a significant difference.

A subset of cancer cells within the tumor population, known as cancer-initiating cells (CICs), is currently considered responsible for cancer recurrence after primary treatment. This subset of cells possesses stem-like capabilities, allowing them to differentiate and re-populate the entire tumor in adverse conditions (including the growth of tumor xenografts in animal models). These cells are also more resistant to different therapies compared with the rest of the tumor population. CICs normally express stemness and EMT markers [29]. As shown in Figure 5, CICs are already present in the HEY cell population and can be isolated through stem-selecting conditioned medium. Under these culture conditions, CICs grow to form small cell aggregates, named “ovospheres” or “spheroids”, which express EMT (N-cadherin, vimentin) and stemness (CD44+) markers that are detectable by immunochemistry (upper panels, Figure 5A). Spheroids, as cancer cells rescued from ascites, also expressed OB-Rb that was detected by immunochemistry and immunofluorescence (upper and lower panels, Figure 5A). CICs also expressed higher levels of EMT (Snail, N-cadherin) and stemness (CD44 and Nanog) marker proteins compared with the non-selected population when probed by immunoblotting (Figure 5B). CICs were also more resistant to a drug commonly used in ovarian cancer treatment (e.g., carboplatin) compared with the whole population (WP). As shown in Figure 5C, less PARP cleavage (an apoptosis marker) was observed in CICs exposed to 50 μM carboplatin compared with the non-selected population. Recently, it has been found that leptin contributes to the maintenance of cancer stem-like cells in breast cancer cells [30]. Thus, we explored whether leptin contributes to the maintenance of the CIC phenotype in HEY cells and favors metastatic potential. As shown in Figure 5D, leptin increased the mRNA levels of the markers for stemness (Oct4 and CD44) and EMT (Zeb2, Snail, and N-cadherin) between 1- and 2-fold compared with the control as measured by real time PCR. A similar increase in the protein levels for some of these markers (N-cadherin, Oct-4, Snail, and CD44) was also found by immunoblotting (Figure 5E). Interestingly, we also observed an increase in OB-Rb expression in HEY spheroids upon leptin incubation, particularly in cells located at the spheroid surface (see Supplementary Figure 5A and 5B). To assess the leptin effect on spheroid formation, we cultured HEY cells in stem-selecting conditioning medium supplemented with different leptin concentrations, similar to those found in obese patients. We also tested the effect of an ascites containing different leptin levels. We chose an ascites sample collected from an overweight cancer patient with high leptin levels (above 10 ng/ml) and another from a healthy BMI patient with low leptin levels (under 5 ng/ml). Next, we incubated CICs with a selecting-medium/ascites mix (50:50 ratio). As shown in left upper and right panels in Figure 5F, adding leptin or ascites to the medium significantly expedited the spheroid formation of HEY cells compared with the control conditions. More importantly, media enrichment with leptin or ascites both prompted faster Matrigel invasion of the cancer cells (left lower panel, Figure 5F). Finally, we assessed the effect of adding ascites with high and low leptin levels on spheroid formation in a primary tissue culture established from a patient with healthy BMI. As shown in Supplementary Figure 5C, supplementing the medium with ascites containing either low or high leptin levels increased the rate of spheroid formation compared with the control conditions (after a 1-week incubation). In addition, the ascites with high leptin levels also increased the size and density of the spheroids compared with the ascites containing low leptin levels. A more intriguing finding was that the isolated cancer cells began to adhere to a surface with low attachment properties upon exposure to ascites with high leptin levels (see Supplementary Figure 5C).

### Concurrent high leptin and OB-Rb mRNA expression in serous ovarian cancer patients from the TCGA database is associated with a worse overall survival rate

**Figure 6:**
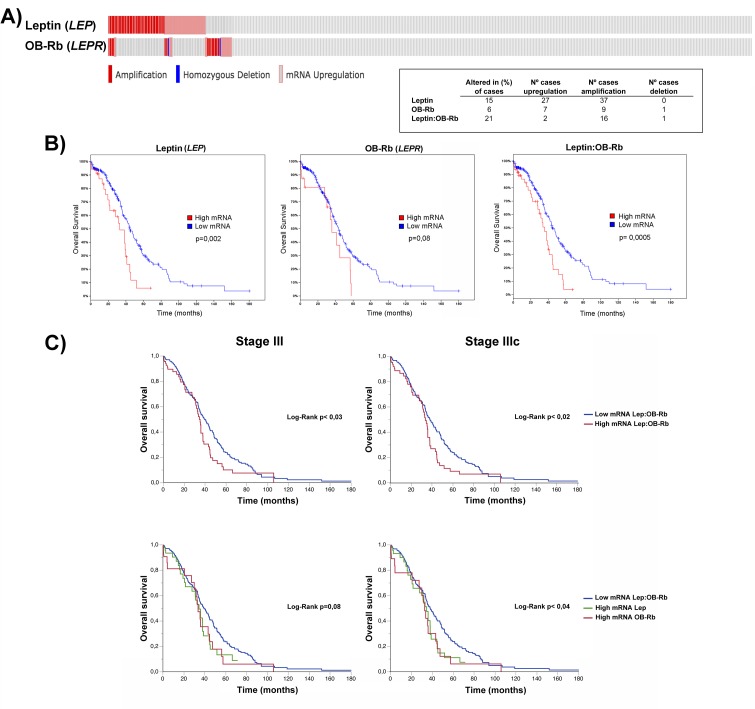
Effects of high OB-Rb and leptin expression on the survival of the serous ovarian cancer TCGA cohort **A.** Oncoprint of leptin and OB-Rb genetic alterations (*upper graphic*; red bars stand for amplification, blue bars for homozygous deletion, and gray bars for gene upregulation) and the distribution table of the specific alterations (*lower right panel*) found in the leptin (*LEP*) and OB-Rb (*LEPR*) genes in the TCGA cohort. **D.** Overall survival curves in patients expressing high versus low levels of leptin (*left graphic*), OB-Rb (*middle graphic*) and leptin:OB-Rb (*right graphic*) mRNAs. **E.** Overall survival curves for all stage III and stage IIIC patients in the high leptin:OB-Rb group mRNA (*upper panels*) and high leptin mRNA or high OB-Rb mRNA subgroups (*lower panels*) compared with the low leptin:OB-Rb mRNA population.

To externally validate the adverse effects of high leptin levels on survival, we downloaded the serous ovarian cancer TCGA database and analyzed it using the bioinformatics tools provided at cBioPortal website. Advanced-stage ovarian cancers are predominant (90%), but not exclusive, in this database. We first evaluated the gene alterations in leptin and OB-Rb in 412 samples included in the RPPA data and 262 in the mRNA Seq V2 data. As shown in Figure 6A, approximately 20% of ovarian cancers (110 cases, 90% in stage III-IV) exhibited alterations (mRNA upregulation or amplification) in either leptin or OB-Rb levels (using an mRNA expression threshold Z-score±1 and a protein/phospho-protein expression threshold Z-score±2). Upregulation and amplification of the leptin gene (*LEP*) correlated with a slight increase in leptin mRNA abundance (see upper panel, Supplementary Figure 6A). In contrast, the upregulation and amplification of the OB-Rb gene (*LEPR*) correlated with a significant increase in OB-Rb mRNA abundance (see lower panel, Supplementary Figure 6A). We also found a significant tendency towards co-occurrence of alterations in this pair of genes by mutual exclusivity analysis (*p* = 0.003). In the high leptin/OB-Rb subgroup, multiple changes in protein levels (123 proteins) and phosphorylation (43 proteins) were found. Among the signaling pathways affected by the higher expression of leptin and OB-Rb were the pathways responding to cytokines, growth factors and hypoxia stimuli (e.g., JAK, STAT3, HIF1α, SNX), as well as those regulating the cell cycle, cell growth and differentiation, the stress response, apoptosis, lipoprotein metabolism and oncogenic transformation (e.g., MAPK, AKT, PTPN, AMPK). Details on the network interactions are shown in Supplementary Figure 6B, and the top five proteins exhibiting changes in protein levels or phosphorylation are shown in Supplementary Figure 6C. Then, we performed a survival analysis, comparing the subgroup expressing higher levels of leptin, OB-Rb or both mRNAs with the rest of the cohort (expressing average or lower levels of both genes), including the complete cohort included in the provisional TCGA dataset (approximately 599 serous ovarian cancers, stage I-IV). As shown in the left middle panel of Figure 6B, high leptin mRNA expression was associated with significantly lower overall survival (log-rank, *p* = 0.02). A tendency towards decreased overall survival was also observed in the subset expressing higher OB-Rb (center middle panel Figure 6B, log-rank, *p* = 0.08). Finally, the subset expressing both higher leptin and higher OB-Rb also showed significantly lower overall survival (right middle panel Figure 6B, log-rank *p* = 0.003). No significant changes were found in the progression-free survival curves comparing the different subgroups with the rest of the cohort. Then, we performed a more precise survival analysis that was restricted only to stage III cases where the achievement of optimal debulking (ideally at the microscopic level or R0) constitutes a major determinant of prognosis. As shown in Figure 6C, ovarian cancers expressing high levels of leptin or OB-Rb mRNAs have a worse overall survival, particularly in stage IIIC patients (81 of 419 cases).

## DISCUSSION

To date, the evidence linking obesity with epithelial ovarian cancer has been controversial. It appears that obese women will have either a higher risk of ovarian cancer or will experience a worse prognosis [4, 5, 31, 32]. Recently, Olsen et al examined the association between high BMI and ovarian cancer risk through a pooled analysis of 15 case-control studies from the Ovarian Cancer Association Consortium. The authors found out that a high BMI (all time points) was associated with an increased risk of type I ovarian cancer (e.g., serous borderline and low-grade serous invasive tumors) [31]. No association was found with type II or high-grade serous ovarian cancers. In terms of survival, Previs et al identified a high BMI as a detrimental factor for survival in low-grade serous ovarian cancers [33]. Similarly, Protani et al performed a meta-analysis of 14 studies and showed a slightly poorer survival rate among obese women than in non-obese women, which was independent of the time when this condition was present during the course of the disease [5]. In the same way, Bae et al pooled 17 cohort studies and found out that being obese at young age and/or 5 years before ovarian cancer diagnosis were associated with a poorer prognosis [32]. However, contradictory to previous studies, these authors found that obesity at the time of diagnosis did not affect survival. Only being underweight at the time of diagnosis was detrimental. Moreover, obese serous cancer patients experienced a better survival compared with underweight patients when optimal debulking was achieved. Pitfalls to explain the contradictory results between studies include differences in the inclusion criteria (e.g., stage, histology, achievement of optimal debulking) determining the intra- or inter-study heterogeneity and the use of different BMI cutoff points to stratify the underweight, healthy and overweight/obese population. Here, we identified overweight and obesity as a negative risk factor for progression-free and overall survival using a selected cohort of stage III-IV, mainly high-grade serous ovarian cancers (see Table 1). The sample size in each group could constitute a weakness of our study. However, the statistical similarity between groups in terms of optimal debulking (either upfront or after neoadjuvant therapy), completion of platinum-based chemotherapy, access to further chemotherapies or secondary cytoreduction and a complete follow-up give our study strength. To our knowledge, this is the first study addressing the role of obesity in high-grade serous ovarian cancer in Latin-American women, a primarily Hispanic population. We also used different methods to confirm the effect of obesity in the cohort, including the study of BMI as an ordinal (based on the World Health Organization classification for healthy, overweight and obesity) or continuous variable in the survival analyses. More importantly, we found that being overweight at diagnosis or after treatment completion constitutes a negative risk factor for disease-associated overall survival. Particularly for high-grade serous ovarian cancers that are sensitive to chemotherapy, maintaining an overweight status or significantly increasing BMI during treatment appears to be detrimental and were independent of achieving an upfront optimal debulking (defined as residual disease less than 1 cm, or inclusive < 5 mm, as cutoff point). Interestingly, overweight or obesity appeared to not have a negative effect in Cox modeling of survival only in the patients where R0 (microscopic residual) was achieved. Thus, we postulated that the residual disease in patients suffering overweight constitutes a negative risk factor that could facilitate recurrence and jeopardize the long-term outcome.

Obesity is characterized by a chronic low-grade inflammation status, where different cytokines, including leptin, circulate at higher serum levels compared with a healthy weight status [34]. Additionally, it has been demonstrated that high circulating leptin levels in obese women with polycystic ovarian syndrome promote OB-Rb expression in luteinized granulosa cells. This effect, in turn, alters endogenous hormone production and exposes the ovarian and fallopian epithelia to an unbalanced hormonal and inflammatory microenvironment, a condition with greater susceptibility to malignant transformation [35–37]. As mentioned in the introduction, two studies have shown that high circulating leptin levels or high OB-Rb expression in the primary tumor correlate with poorer outcomes in ovarian cancer [18, 19]. Here, we quantified OB-Rb expression by immunohistochemistry in samples obtained from primary tumors (type I and II serous cancers), ascites and metastatic lesions (mainly type II serous cancers). Most of the samples originated from overweight patients. Using a color deconvolution method to digitally score the OB-Rb staining, we were able to objectively categorize the staining intensity in four categories (weak or + to the strongest intensity or ++++), independent of the observer bias. First, we found that OB-Rb is more intensely expressed in type I ovarian cancers (borderline serous) compared with the benign serous counterparts. We also found a significant change in the staining intensity histogram when comparing the primary tumor with the ascites cancer cells and the metastatic lesions, all collected from high-grade serous ovarian cancer cases [38]. In fact, the OB-Rb staining intensity histogram was clearly skewed towards the weakest intensity category in the primary lesions, while it was homogeneously distributed among the four categories in the metastatic lesions. Similarly, ascites cancer cells strongly express OB-Rb as detected by immunochemistry. Thus, both ascites and the metastatic lesions showed a similar pattern to the type I tumors (see Figure 2). Because the level of OB-Rb expression in the primary tumor did not necessarily correlate with OB-Rb expression in the metastatic sites or the ascites, we suggest analyzing all tumor sites before assigning a staining score to a particular case and associating its OB-Rb expression with prognosis. In addition, we postulate that this differential OB-Rb expression could be influenced by the cancer cells’ surrounding microenvironment. In this sense, we postulate that obesity could enrich the tumor microenvironment with cytokines or adipokines (e.g., leptin) that negatively influence cancer cell behavior. In this respect, Matte et al have shown that high ascites leptin levels ( > 658 pg/ml) are also associated with a worse outcome in a univariate survival analysis [39]. Therefore, we measured serum and ascites leptin levels by ELISA in samples obtained from healthy BMI and overweight high-grade serous ovarian cancer patients. We also searched for any correlation between serum and ascites leptin levels. As shown in Figure 2, healthy BMI women undergoing surgery for benign or malignant conditions show similar circulating leptin levels (8.1±2.5 *vs*. 6.7±2.1 ng/ml, respectively, *p* = NS), which were equivalent to previously reported levels [34]. We also found significantly higher circulating leptin levels among overweight cancer patients (25.7±3.1 ng/ml) compared with healthy BMI patients. More importantly, circulating leptin levels positively correlated with the ascites levels. Overweight and obese patients had higher ascites levels compared with healthy BMI cancer patients. Therefore, high leptin levels, and perhaps high levels of other cytokines (e.g., IL-6), in obese patients could contribute to the maintenance and survival of the dormant cancer cells that remain after debulking surgery (e.g., floating cells in the ascites or abdominal cavity), facilitating their settlement in new secondary locations (e.g., adipose tissues or mesothelium at the peritoneum) and the reappearance of recurrent disease [8, 39, 40]. In this sense, and despite the fact that we did not utilize serum or/and ascites samples from our entire cohort to measure the leptin levels, we were able to analyze five matched serum/ascites cases with adverse outcomes. These cases experienced either an earlier recurrence or cancer-related death. Four cases corresponded to overweight/obese patients, all with high leptin levels. The fifth case corresponded to a patient with healthy BMI at diagnosis. However, this patient had a previous history of bariatric surgery that was performed the year before the cancer diagnosis. Currently, we are recruiting new patients, registering their BMI and collecting matched serum and ascites samples to confirm our preliminary finding.

To directly explore the biological effects of leptin and OB-Rb levels in ovarian cancer cells, we chose to use cell lines and primary tissue cultures representing the two types of serous ovarian cancer. We also used cell lines expressing different OB-Rb levels and tested the effect of different leptin concentrations. Based on the serum and leptin levels found in our overweight cohort and the levels reported by others [41], we selected 100 ng/ml for additional experiments.

Previously, Choi et al have shown that high leptin concentrations (100-1000 ng/ml) induce cell proliferation in BG-1 ovarian cancer cells using [^3^H] thymidine incorporation assays. This group also demonstrated that the increase is mediated by MAPK activation, because PD98059 addition (MEK inhibitor) completely reverses leptin-induced cell growth [14]. Here, we confirmed that leptin indeed increased cell proliferation in ovarian cancer cells, but only in the OB-Rb high-expressing cancer cells (A2780, UCI 101). We also found that high leptin doses (above 100 ng/ml) and longer incubations (up to 72 h) were required to observe its effect on cell proliferation (see Supplementary Figure 3).

As reported for other epithelial cancers (e.g., colon cancer), leptin stimulates migration and invasion by binding to OB-Rb [16, 42]. Using high leptin concentrations, the magnitude of the effect is quite similar in all ovarian cancer cell lines tested, despite the differences in receptor expression. In fact, leptin's effects on cell migration and invasion were observed in both low-OB-Rb-expressing (e.g., SKOV3) and in middle- or high-expressing (e.g., HEY and Asc3 primary tissue culture) cancer cells using wound-healing and Boyden chamber assays (see Figure 3). Leptin's effects were observed to be independent of the p53 status of the ovarian cancer cell, because both p53 wild type (HEY) and p53 null (SKOV3) cell lines exhibited increased cell migration and invasion upon leptin exposure. These findings support a negative effect of the higher circulating leptin levels observed in obesity for both type I and type II epithelial ovarian cancers.

Normally, OB-Rb present on the cell membrane is cyclically internalized to a cytoplasmic pool, independent of ligand binding, and later recycles to the surface [43, 44]. Upon leptin binding, membrane OB-Rb receptor levels are significantly decreased, as measured by biotinylation assays. By blocking binding with a leptin-neutralizing antibody, we confirmed that leptin binding to OB-Rb is required for the increase in cell migration and invasion observed in the ovarian cancer cell lines. In fact, pre-incubation with this antibody nearly completely abrogated the leptin-induced increase in cell migration observed in the wound-healing assays (see Figure 3). Upon leptin binding, we observed changes in protein levels and phosphorylation of different kinases and small GTPases, including JAK/STAT3, MAPK, AKT, mTOR, RhoA/ROCK and MYPT1, all key players for cell growth and migration in other cell types [21, 45–47]. Furthermore, pre-incubation with selective inhibitors that act at different levels of the cascade, such as AG490 (JAK inhibitor), LY294002 (PI3K inhibitor) or fasudil (ROCK inhibitor), all nearly completely abrogated leptin-induced cell migration and invasion in the different assays performed here. Only UO126 (MAPK inhibitor) did not affect leptin-stimulated cell migration, suggesting that this kinase is not involved in this leptin effect (data not shown; see Figure 3, Supplementary Figures 3 and 4).

Upon leptin exposure, a greater number of lamellipodial protrusions were observed at the edges of the wound, reflecting cell movement stimulation and a faster wound-healing rate. Using a higher magnification, it was also possible to identify stress-fiber assembly (F-actin polymerization) in the lamellipodia of most of the migrating cells and the formation of new focal adhesion complexes (a result of integrin contact with the extracellular matrix and interaction with FAK, vinculin and PI3K; see Figure 4). Thus, it is possible to hypothesize that leptin would act as a cell movement stimulus for ovarian cancer cells, as described for other cytokine family members, growth factors and extracellular matrix components [21, 48]. Taken together, our results demonstrate that leptin mediates the aggressiveness of epithelial cancer and offer an explanation as to why ovarian cancer patients with higher circulating serum leptin levels or leptin receptor-expressing tumors experience a worse survival rate. Importantly, our results support a linkage between obesity and ovarian carcinogenesis, in which leptin can be a key player.

It is widely accepted that a subset of cancer cells expressing EMT and stemness markers within tumors, referred to as CICs, adapt and respond more efficiently to environmental stimuli. CICs are generally more resistant to chemotherapy, as shown for our isolated HEY CICs and carboplatin. Upon stimulation, this subset of cells is more likely to migrate and invade, promoting cancer progression [25]. Similar to reports in breast cancer cells, we observed leptin-induced expression of EMT genes and stemness markers in CICs isolated from ovarian cancer (CD44+), supporting its role in the maintenance of a more aggressive phenotype [49]. In fact, HEY CICs tend to form spheroids more rapidly and to be more invasive in Matrigel upon leptin stimulation compared with the non-selected population (see Figure 5). Interestingly, we also observed an increase in OB-Rb expression within the spheroids upon leptin addition, suggesting a self-reinforcing signaling module (see Supplementary Figure 5). This finding is in accord with the leptin-induced effect in granulosa cells and liver CICs [35, 50] and also offers an explanation for the tendency for the co-occurrence of high mRNA leptin and OB-Rb levels in the TCGA serous ovarian cancer patients. These data also suggest potential defects in the intracellular trafficking leading to altered OB-Rb cell surface expression and abnormal functionality and response to the ligand [43, 44]. Finally, to assess the effect of low and high ascites leptin levels, we supplemented the stemness selection medium with the ascites collected from obese and healthy BMI cancer patients. We observed a significant increase in the speed of spheroid formation and the number and size at 8 days of incubation in the ascites-supplemented wells, either with high or low ascites leptin levels, compared with the non-supplemented wells (see Figure 5 and Supplementary Figure 5). This effect was more significant with high ascites leptin levels. More interestingly, we observed the attachment of isolated cancer cells in the wells supplemented with ascites containing high leptin levels, despite being grown on a low-adherence surface. These findings support the role of leptin and other mediators present in the ascites in contributing to the maintenance of the CIC phenotype and promoting the settlement and formation of metastatic foci (e.g., in the peritoneum or omentum).

To externally validate the association between high leptin and OB-Rb levels and poor outcome, we downloaded the TCGA serous ovarian cancer database (including mutations, putative copy-number alterations from GISTIC, mRNA expression data and protein/phospho-protein levels) and analyzed the data using the cBioPortal tools [51, 52]. First we searched for genetic alterations in both genes. We found that approximately 20% of serous ovarian cancers present with alterations in at least one of the genes. For the leptin gene (*LEP*), the gene alterations correspond to gene upregulation and amplification (using a Z-score ±1 threshold with respect to the average mRNA abundance in the complete cohort). For the OB-Rb gene (*LEPR*), the alterations also correspond to upregulation and amplification. Only one *LEPR* mutation was found (K1154N) that translates to gene amplification (see Figure 6). By plotting the putative copy-number alterations (CNA) and mRNA expression levels for *LEP* and *LEPR*, we found that the upregulation or amplification of the *LEP* gene in ovarian cancer correlates with a slight increase in the *LEP* mRNA levels in the samples analyzed by RNA Seq V2 in the TCGA database. In contrast, the upregulation and amplification of *LEPR* resulted in a significant increase in the *LEPR* mRNA levels. We also found that ovarian cancers with high mRNA *LEP*/*LEPR* levels exhibit multiple protein changes (either in level of expression or phosphorylation status) involving different signaling pathways, some of which are directly related to the response to cytokines, growth factors and hypoxia stimuli (e.g., JAK, STAT3, HIF1α, SNX), cell cycle regulation, cell growth and differentiation, the stress response, apoptosis, lipoprotein metabolism and oncogenic transformation (e.g., MAPK, AKT, PTPN, AMPK; see Supplementary Figure 6). More importantly, several of these kinases (e.g., JAK, STAT3, MAPK, and AKT) are directly involved in leptin-induced cell migration and invasion in other epithelial cancers [16, 42, 46]. Finally, we performed a survival analysis comparing this subgroup with the rest of the TCGA cohort. We found that high *LEP* mRNA levels alone or high *LEP*/*LEPR* mRNA levels are associated with poorer overall survival. For high *LEPR* mRNA levels alone, we found a non-significant tendency towards lower overall survival (see Figure 6). Taken together, our findings suggest that OB-Rb overexpressing tumors may require lower leptin levels to trigger the different pathways and to negatively impact survival [18]. In other hand, low or average OB-Rb-expressing tumors in the presence of higher leptin levels, either from autocrine or paracrine origin (e.g., adipose tissue in obese patients), can also trigger these pathways and lead to the same final adverse effect. Therefore, it is possible to infer that the high leptin levels present in the microenvironment (e.g., ascites, omentum or mesothelium) could also negatively impact the outcome of advanced tumors with low OB-Rb expression, emphasizing the role of an overweight status in the prognosis of ovarian cancer. Unfortunately, the TCGA database did not provide BMI information to correlate overweight, *LEP* and *LEPR* expression levels and the survival curves in this cohort.

Two recent animal model studies support our findings and the role of leptin and obesity in high-grade serous ovarian cancers. First, Makowski et al demonstrated significant differences in gene expression and metabolomics profiling between the ovarian tumors from the obese versus non-obese mice in a genetically engineered mouse model of serous ovarian cancer, similar to our findings in the high leptin/OB-Rb mRNA subgroup from the TCGA database. Interestingly, ovarian tumors were significantly larger and more widely spread in the obese mice in this study [53]. Second, Al-Wahab et al have recently showed that high-energy balance (HED) or caloric excess is a tumor-promoting factor in an isogeneic immunocompetent mouse model of epithelial ovarian cancer. Mice on an HED diet displayed the most extensive tumor formation with the highest tumor score at all organ sites. This widely spread disease was accompanied by increased levels of multiple growth factors, cytokines (e.g., IL-6) and leptin. Immunohistochemistry analysis of the tumor samples from the HED mice also demonstrated increased activation of AKT and mTOR [54].

Initially, we hypothesized that blocking leptin binding to OB-Rb could be an attractive and potentially effective target therapy for obese cancer patients with high circulating leptin levels. However, the preliminary results from our group suggested that adding a leptin-blocking peptide is not sufficient to abrogate the ascites-induced spheroid formation and invasiveness. A potential explanation for its ineffectiveness is the presence of other pro-inflammatory cytokines in the ascites. In fact, we have recently discovered that high leptin levels stimulate the secretion of other cytokines (e.g., IL-6) both from cancer cells and from other cell types present in the ascites (e.g., macrophages; manuscript in preparation). The partial response to a leptin-blocking peptide is in agreement with Coward and colleagues’ finding that siltuximab treatment (an anti-IL6 antibody) significantly reduces tumor burden, but does not completely eliminate metastatic foci and ascites accumulation in xenograft mouse models [55].

Multiple cytokines are found in the ascites of cancer patients [39], and many are likely circulating at higher concentrations in the obese population. This cytokine profile reflected the chronic inflammatory state induced by obesity that promotes cancer progression. Different mechanisms have been proposed to explain the link between obesity and cancer. A universal convergence mechanism involves over-activating the nutrient-sensing mTOR pathway in both normal and cancer cells [56]. Activation of mTOR promotes obesity, and obesity, in turn, activates mTOR. This pathway has been involved not only in malignant transformation but also in growth, proliferation and metastasis in ovarian cancer [57]. Here, we demonstrated that high leptin levels, as observed in the obese population, indeed activate the PI3K/AKT/mTOR signaling pathway in ovarian cancer cells (e.g., SKOV3 and UCI101). Therefore, we hypothesize that therapies targeting mTOR activity should be effective against ovarian cancer, particularly in obese patients. Caloric restriction, rapamycin, metformin, and statins, all inhibit the mTOR pathway [56, 58]. Particularly, the intra-peritoneal administration of rapamycin has shown to significantly reduce BMI and leptin levels in mice on a high-fat diet [59]. Therefore, all of these potential therapies should be tested either alone or in combination with chemotherapy in ovarian cancer, particularly in obese patients.

In conclusion, we present clinical and molecular evidence supporting the adverse effect of overweight status and obesity in high-grade serous ovarian cancer. We also demonstrated that the abnormal activation of the leptin/OB-Rb pathway is a major determinant of the worse outcome in obese women. Further studies must be conducted to explore the impact of changing the diet (caloric restriction), promoting exercise or using statins or metformin in the prognosis of the overweight and obese ovarian cancer population [54, 60, 61].

## MATERIALS AND METHODS

### Ethics statement

Investigation has been conducted in accordance with the ethical standards and according to the Declaration of Helsinki and according to national and international guidelines and has been approved by the authors’ institutional review board.

### Reagents

Recombinant human leptin and AG490 were purchased from Sigma-Aldrich Corp. (St. Louis, MO, USA). UO126 and LY294002 were purchased from Cell Signaling Technology (Boston, MA, USA). Fasudil was purchased from Abcam (Cambridge, MA, USA) and the leptin-neutralizing antibody was purchased from R&D Systems (Minneapolis, MN, USA).

### Cell line maintenance and isolation of cancer-initiating cells (CICs)

The ovarian cancer lines, SKOV3, UCI101, A2780, OVCAR-3 and HEY, were maintained in RPMI supplemented with 10% fetal bovine serum (FBS; Invitrogen^TM^, Life Technologies^TM^, Grand Island, NY, USA) plus antibiotics/antimycotics (Invitrogen^TM^). The cell lines were authenticated using the Genemarker 10 kit (Promega, Madison, WI, USA) and routinely screened for mycoplasma infection by PCR. For protein and RNA experiments, the cells were plated at 50% confluence in 10-cm^2^ and 6-cm^2^ tissue culture dishes, respectively. Twenty-four hours before treatment, the medium was removed and changed to medium supplemented with 5% charcoal-treated FBS. CICs were isolated from the HEY cells (2.5×10^4^ cells per well) using a stem-cell selecting media (DMEM/F12 medium; Invitrogen^TM^) supplemented with epidermal growth factor (EGF; 20 ng/ml), fibroblast growth factor-2 (FGF-2; 10 ng/ml), bovine serum albumin (BSA; 0.4%) and insulin (5 μg/ml) without FBS under non-attachment conditions (Corning^®^ Costar^®^, Manassas, VA, USA). After 6 days, spheroids containing CICs with a diameter between 40 and 70 μm were collected.

### Patient selection

Seventy patients who were diagnosed with stage III and IV epithelial ovarian cancer between 2006 and 2013 were selected from database at our institution. The majority of patients underwent primary debulking surgery. In some patients who were not fit for upfront surgery, the initial neoadjuvant chemotherapy was followed by interval debulking surgery. Progression-free survival was calculated from the date of surgery for the patients undergoing surgery or from the biopsy (CT-guided needle biopsy or taken during diagnostic laparoscopy) in those undergoing neoadjuvant therapy. To calculate the overall survival, we obtained the death certificate (including cause of death) issued by the Chilean civil registration for all of the cases. We initially divided the cohort based on BMI, considering healthy weight a BMI ≥18.5 to < 25 Kg/m^2^ and overweight ≥25 Kg/m^2^. We also analyzed BMI as continuous variable or stratified in four ordinal categories ( < 18.5, ≥18.5- < 25, ≥25- < 30, and ≥30 Kg/m^2^). The clinical demographics of the study cohort are summarized in Table 1. The Institutional Review Board (IRB) at our university approved the study.

### Establishment of primary tissue cultures from human epithelial ovarian cancer specimens

The primary tumor or ascites samples were obtained from patients with advanced stage serous-papillary ovarian carcinoma who were undergoing primary surgery at our institution that had previously provided signed informed consent (IRB-approved protocol). The establishment of primary human ovarian tissue cultures was performed according to previously described protocols [62]. Briefly, the tumor samples were incubated in digestion medium (Hank's balanced salt solution; HBSS) containing 2 mg/ml collagenase (Sigma-Aldrich Co.) for 30 min at 37°C with agitation. The digested mixture was then filtered through a 50-μm pore nylon mesh (PGC Scientific, MD, USA) to separate the cells from the stromal fraction. The epithelial cells retained by the filter were washed, re-suspended in culture medium (DMEM/F12 supplemented with 10% FBS plus antibiotics/antimycotics) and transferred to tissue culture dishes. The ascites (50 ml) was centrifuged, the pellet was re-suspended in a 1:1 solution of the ascites supernatant and DMEM/F12 with 10% FBS and plated in tissue culture dishes containing culture medium.

### RT-PCR /real-time PCR

Total RNA was isolated from the ovarian cancer cell lines and primary ovarian tissue cultures using TRIzol (Life Technologies^TM^). The cDNAs were generated using reverse transcriptase (Superscript II; Invitrogen^TM^). Semi-quantitative PCR reactions were performed with cDNAs generated from the cell lines and primary cultures using Taq polymerase (Invitrogen^TM^) and leptin receptor primers (sense, 5′-CAG AAG CCA GAA ACG TTT GAG -3′ and antisense, 5′-AGC CCT TGT TCT TCA CCA GT -3′). Primers amplifying a region of glyceraldehyde-3-phosphate dehydrogenase were used in parallel as an internal control to test the integrity of the starting cDNA.

Real-time PCR reactions were performed using the cDNAs generated from cancer-initiating HEY cells with the same RT-PCR protocol described above. The cells were treated with leptin alone (100 ng/ml for 6 h) or with a 24-h pre-incubation of vehicle or simvastatin (1 μM). The mRNA expression levels of Zeb2, Snail, cdh2 (N-cadherin), Oct-4 and Hrpt1 (housekeeping gene) were measured by real-time PCR (qRT-PCR) using the primers described in Supplementary Table 1. The qRT-PCR reactions were performed using the SYBR Green Master Mix (Applied Biosystems^®^, Life Technologies^TM^) in an ECO Real-Time PCR System (Illumina, Inc., San Diego, CA, USA). A melting curve analysis was performed on each sample after the final cycle to ensure that a single product was obtained. The relative amounts of all mRNAs were calculated using the 2^−ΔΔct^ method [63].

### Immunoblotting

After treatment with leptin or the control, the cells were harvested in cold PBS, and the pellet was re-suspended in lysis buffer (20 mM Tris-HCl, pH 7.4, 137 mM NaCl, 2 mM EDTA, 1% Triton X-100, 10% glycerol) for 20 min at 4ºC. Next, the lysate was sonicated and centrifuged at 14,000 *g* for 20 min at 4°C, after which, the supernatant was collected. The protein concentrations were determined using the Bradford assay. A total of 50-100 μg of the protein extract was loaded in each lane, separated on a 10% SDS-PAGE gel, transferred to nitrocellulose membranes and incubated overnight with the following primary antibodies: leptin receptor (1:1000) and CD44 (1:1000) (Santa Cruz Biotechnology, CA, USA); phospho-STAT3 (1:1000), MAPK (1:1000), phospho-MAPK (1:1000), phospho-AKT (1:1000), mTOR (1:1000), phospho-MYPT1 (1:1000), RhoA (1:1000), PARP (1:1000), and Snail (1:1000) (Cell Signaling Technology), Nanog (1:1000) (R&D Systems); and N-cadherin (1:1000), E-cadherin (1:500), Oct-4 (1:1000), Nanog (1:1000), Zeb2 (1:1000), Vimentin (1:1000) (Abcam) and β-actin (1:10000) (Sigma-Aldrich Corp.). Peroxidase-conjugated goat anti-mouse/rabbit IgG secondary antibodies (1:3000, Bio-Rad Labs, CA, USA) were applied for one hour at room temperature (RT). The reaction was developed with chemiluminescence using the ECL Western blot analysis system (NEN, Western lightning, Perkin-Elmer).

### Immunohistochemical detection of OB-Rb and digital scoring

Paraffin blocks of the serous and mucinous lesions (benign, borderline, malignant and metastatic) were obtained. OB-Rb immunohistochemistry (DAB, hematoxylin) was performed on 5-μm paraffin slices of the tumor samples, and the data analysis was performed by digitally measuring the DAB intensity in 40X images using ImageJ software. DAB staining was scored in 320 microscopic fields from 10 samples of each tumor condition using a symbol scale: +, ++, +++, and ++++ (representing 25%, 50%, 75% and 100% of the stronger DAB staining, respectively).

### Immunofluorescence protocols

For stress-fiber, lamellipodia and focal adhesion complex experiments, a total of 20,000 cells/per well were seeded in a 24-well plate (Nunc^®^) containing glass cover slips that were previously treated with fibronectin and RPMI. SKOV3 cells were treated with leptin (100 ng/ml) for 6 h prior to fixation. The cells were washed with PBS, fixed with 4% paraformaldehyde/4% sucrose for 10 min, washed three times with 1X PBS and finally blocked with 4% nonfat dry milk in PBS/0.1% Triton. The primary antibodies were diluted in 1% nonfat dry milk in PBS buffer and used at a dilution of 1:200 for anti-vinculin (Sigma-Aldrich Corp.). The antigen–antibody complex was washed and incubated with a fluorescein 5-isothiocynate-conjugated anti-mouse monoclonal antibody (Invitrogen Molecular Probes, Carlsbad, CA, USA). F-actin (stress-fibers, lamellipodia) was stained with rhodamine-labeled phalloidin (Invitrogen^®^) at a dilution of 1:50. The nuclei were stained with Hoechst at 1:5000, and the sections were mounted with Fluoromount^TM^ (Sigma-Aldrich Corp).

For the spheroid studies, the spheroids were collected and washed 2X in PBS buffer before being fixed in 4% PFA (15 min at RT). After fixation, the samples were washed 2X in PBS buffer before permeabilization with 0.01% Triton-X100 (10 min at RT). After overnight blocking at 4ºC in PBST (0.01% Triton X-100, 3% BSA in PBS), the spheroids were incubated with primary antibodies to either OB-Rb primary (1:100), Vimentin (1:100) or N-cadherin (1:100) for 48 h at 4°C with gentle rotation. Finally, after 4 washes with PBST (30 min each, at 4°C), the spheroids were incubated with a goat anti-mouse secondary antibody conjugated to Alexa-555 (1:1000) for 24 h at 4°C. The cell nuclei were counterstained with DAPI (1:50000) and incubated for 30 min at RT. The spheroids were mounted in a sandwich chamber assembled between a glass slide (1-mm thick) and a coverslip (22×22 mm, No.1), using three layers of double-sided scotch tape as a spacer. After DAPI staining, the spheroids were washed 2X in PBS buffer (10 min each, at RT) and then collected in 4 μl of PBS and allowed to gently adhere to the glass slide. After carefully removing the excess buffer, the spheroids were mounted in 20 μl of Fluoromount-G (Sigma Aldrich Corp.). Finally, the coverslip edges were sealed with nail polish.

### Confocal image acquisition

The images were recorded on an Olympus FV1000 confocal microscope equipped with a 20X/NA 0.75 air objective at 100 μm C.A. DAPI fluorescence was acquired with a 440-nm diode laser, a 405-440-nm dichroic mirror and a 465-495-nm emission filter. Alexa-555 fluorescence was acquired with a 543-nm He-Ne laser, a 488/543/633-nm dichroic mirror and a 560-nm long pass emission filter. The negative control, with no primary antibody added during the immunofluorescence protocol, did not exhibit any detectable fluorescence signal. The DAPI and Alexa-555 signals were merged using ImageJ software.

### MTS assay

To assess leptin-mediated viability, ovarian cancer cells were plated in RPMI at 3000-5000 cells per well in 96-well microtiter plates and incubated for 48 h with different concentrations of leptin (0-1000 ng/ml). Cell viability was assessed using the previously described 3-(4,5-dimethylthiazol-2-yl)-2,5-diphenyltetrazolium bromide; MTS) dye reduction assay [60]. All experiments were performed in quintuplicate and repeated at least five times. The results are presented as the mean±standard error (SE).

### Boyden chamber invasion assay

Transwell inserts containing 8-μm isopore membranes (Nunc^®^, NY, USA) were coated with 50 μl of Matrigel (diluted 1:5; BD Biosciences, San José, CA, USA). Approximately 100,000 cells (A2780, SKOV3 or primary tissue cultures) were suspended in 200 μl of RPMI (or DMEM/F12) with 5% charcoal-treated FBS and spread over the Matrigel. The inserts were placed in a 6-well plate containing RPMI or DMEM/F12 with 10% FBS as a chemo-attractant. The cells were treated with leptin alone (100 ng/ml for 24 h) or pre-incubated for 30 min with vehicle or fasudil (10 μM). To measure the number of cells passing through the insert, immunocytochemistry for pan-cytokeratin (Sigma-Aldrich Corp.) was performed on the filters, which were mounted on cover slides with Kaiser's glycerol gelatin (Merck KGaA, Darmstadt, Germany). The inserts were examined under a microscope (Nikon Eclipse E-200, Nikon Instruments Inc., Tokyo, Japan) to quantify the number of cells per HPF (400X) that degraded the Matrigel and passed through the isopore membrane. In each experiment, 15 fields were counted, and the data are expressed as the mean±SE of four different experiments.

### Wound-healing assay

Cell migration was studied using a wound-healing assay. SKOV3 and HEY cells (100,000 per well) were seeded on 24-well plates containing 500 μl of RPMI medium. The cells were then washed 3 times with PBS, and a wound was generated by removing the cells in the center of the well with a sterile pipette tip. The detached cells were washed away with PBS. The cells were incubated in different experimental conditions for 6 to 24 h. Cell migration was studied by treating the cells with 100 ng/ml of leptin alone or with a 30 min pre-incubation with the leptin-neutralizing antibody (10 μg/ml), AG490 (50 μM), fasudil (10 μM), UO126 (10 μM) or LY294002 (10 μM). Three images were obtained along the wound with a Nikon TMS inverted microscope connected to a Nikon Coolpix 4500 camera (Nikon Instruments Inc.). Wound closure was quantified by measuring the area in pixels between the edges of the wound using a measurement tool in Adobe Photoshop^®^ with a grid superimposed on the image as a guide for the measurements. The wound width was normalized to 100% at 0 h for each treatment condition, and the data represent the percentage of wound closure.

### Pinpoint cell surface protein labeling

The Pinpoint Cell Surface Protein Isolation Kit (Thermo Scientific Pierce, Whaltman, MA, USA) was used to biotinylate and isolate cell surface proteins according to the manufacturer's instructions (Santa Cruz, CA, USA). Briefly, HEY or SKOV cells were stimulated with vehicle or leptin (100 ng/ml) for 30 min. The cells were then washed with ice-cold PBS, and the surface proteins were biotinylated and isolated using immobilized avidin before immunoblotting for leptin receptor and β-actin levels. The result is representative of four experiments performed under the same conditions.

### RhoA pull-down assay

RhoA pull-down assays were performed using the Rho activation assay kit according to the manufacturer's instructions (Cell Biolabs Inc., San Diego, CA, USA). SKOV3 cells at 80-90% confluency were treated with leptin (100 ng/ml) or vehicle for 6 h. After treatment, the cells were washed with ice-cold PBS and lysed with cell lysis buffer. Equal amounts of the protein lysates were used for the pull-down assays with Rhotekin-RBD beads. The pellets were washed and eluted using the buffers included in the kit. After boiling, the samples were separated on an SDS-PAGE gel. The total levels of RhoA and actin were measured by immunoblot analysis of the cell extracts using anti-RhoA (Cell Signaling) and anti-actin antibodies. The results are representative of two different experiments.

### TCGA clinical samples and data analyses

The Cancer Genome Atlas ovarian cancer molecular and clinical data were downloaded from the Broad Institute TCGA Data and Analysis website (https://confluence.broadinstitute.org/display/GDAC/Home), which provides data that have been subjected to standardized preprocessing and normalization, as described in detail on their website. The downloaded data were further analyzed (e.g., mutual exclusivity between genomic alterations, biological pathway exploration, and survival analysis) using the cBioPortal for Cancer Genomics (http://cbioportal.org) [51, 52].

### Statistical analyses

The JMP11 (SAS Institute, Inc., Cary, NC) software package was used for the data analyses. The results are expressed as the mean±SE. Statistical significance was calculated by Student's *t*-test, Chi-squared test or analysis of variance. Survival curves were generated with the Kaplan Meier method and analyzed using the log-rank, Wilcoxon and Cox proportional hazard models. *P*-values less than 0.05 were considered significant.

## SUPPLEMENTARY MATERIAL TABLE AND FIGURES


