# Supplementary material for: Leptin stimulates migration and invasion and maintains cancer stem-like properties in ovarian cancer cells: an explanation for poor outcomes in obese women
Source: Oncotarget. 2015 May 22;6(25):21100–19. doi: 10.18632/oncotarget.4228 (PMC4673253; doi:10.18632/oncotarget.4228)
Supplement: Supplementary file 1 [file oncotarget-06-21100-s001.pdf]

Leptin stimulates migration and invasion and maintains cancer stem-like properties in ovarian cancer cells: an explanation for poor outcomes in obese women

Supplementary Material

Supplementary Table 1: Real-time PCR primers used in this study.

	Sense /Antisense 5'-3'	Size	Annealing T°	Reference	*E*
**Hprt1**	GACCAGTCAACAGGGGACAT / ACACTTCGT GGGGTCCTTTTC	194	60°C	Zhang et al., 2005	1.94
**Cdh2**	TGTGGGAATCCGACGAATG/ GTCATATGGTGGAGCTGTGGG	151	60°C	Wang et al., 2013	2.02
**Zeb2**	AGGCATATGGTGACGCACAA / CTTGAACTTGCGGTTACCTGC	78	60°C	Clarhaut et al.,2009	2.01
**Snail**	CGCGCTCTTTCCTCGTCAG / TCCCAGATGAGCATTGGCAG	181	60°C	Dohadwala et al., 2006	2.06
**Oct4**	TCCCATGCATTCAAACTGAGG/ CCAAAAACCCTGGCACAAACT	104	60°C	Olmer et al., 2010	1.98
**Cd44**	CCCAGACGAAGACAGTCCCT / GCCTCTTGGTTGCTGTCTCA	104	60°C	Unpublished	1.97

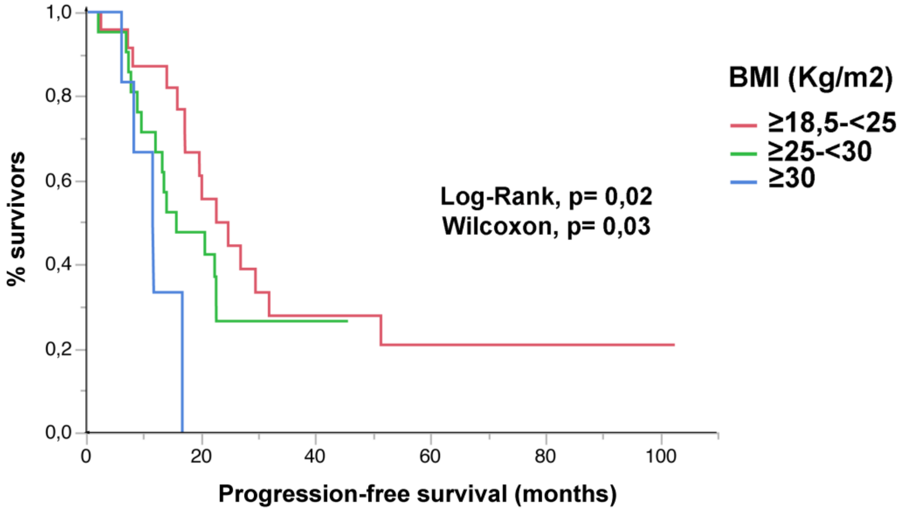

Supplementary Figure 1: Progression-free survival stratified by BMI in high-grade ovarian cancer (healthy (normal): 18.5-<25 kg/m^2, overweight: ≥25-<30 kg/m^2, and obesity: ≥30 kg/m^2).

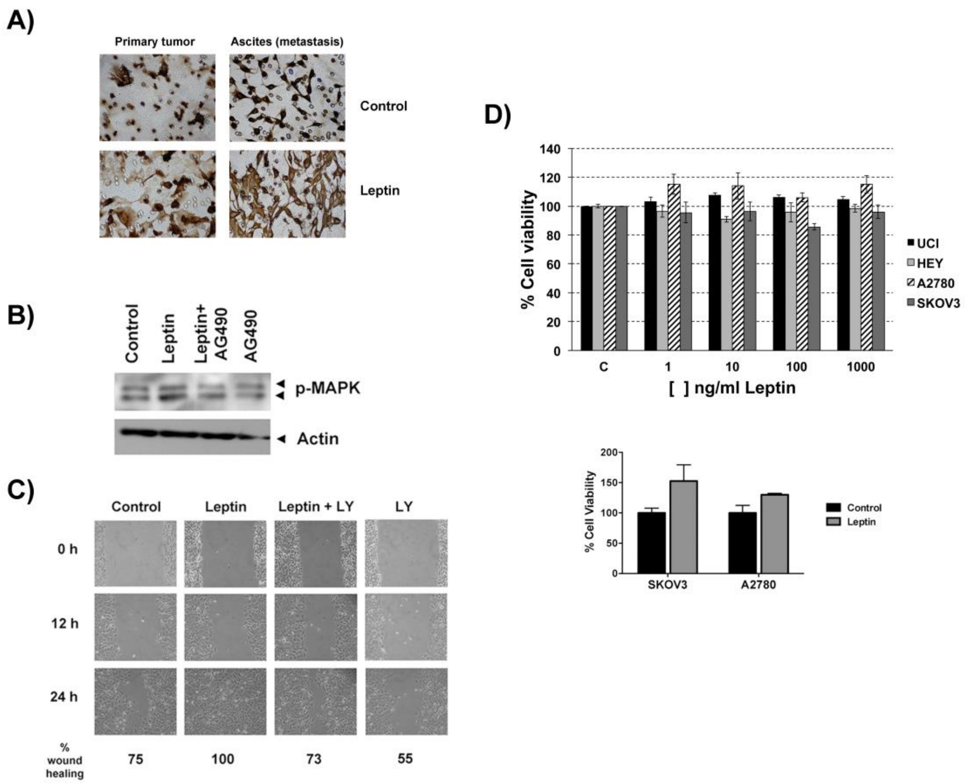

Supplementary Figure 3: A) Effects of leptin on cell invasion in primary tissue cultures established from the primary tumor and ascites collected from an advanced serous ovarian cancer. B) Effects of AG490 (JAK inhibitor) on leptin-induced phosphorylation of a downstream kinase (MAPK) in HEY cells. C) Effect of the PI3K inhibitor, LY294002, on the migration of HEY cells as measured using a wound-healing assay. D) Effects of leptin on cell proliferation of different ovarian cancer cell lines (UCI 101, HEY, A2780, and SKOV3; 1-1000 ng/ml dose and 24-72-h time course).

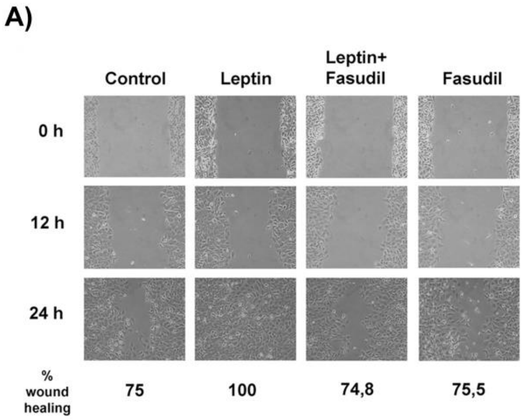

Supplementary Figure 4: Fasudil effects on leptin-mediated cell migration in SKOV3 cells.

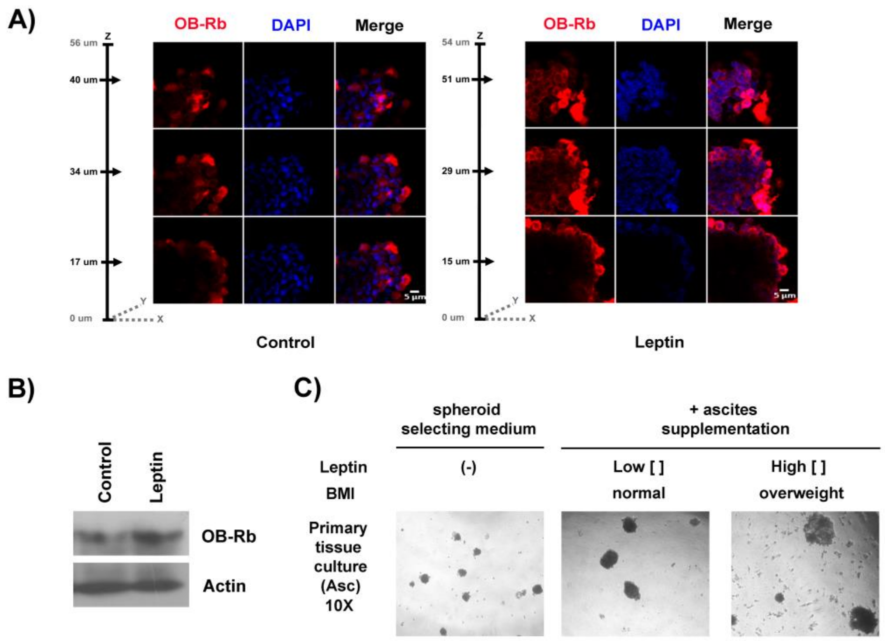

Supplementary Figure 5: OB-Rb expression was measured in the outer and inner layers of the HEY spheroids treated with vehicle or leptin (100 ng/ml for 24 h) by immunofluorescence with confocal microscopy (A) and immunoblotting (B). C) Effect of supplementing the stem-selecting media with ascites containing high or low leptin levels on spheroid formation.

A)

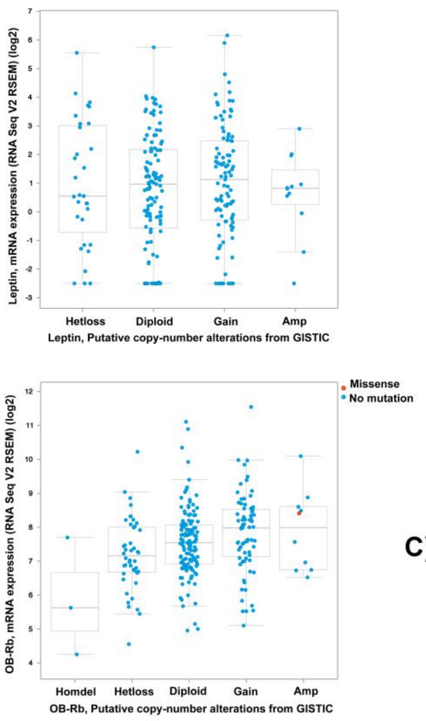

B)

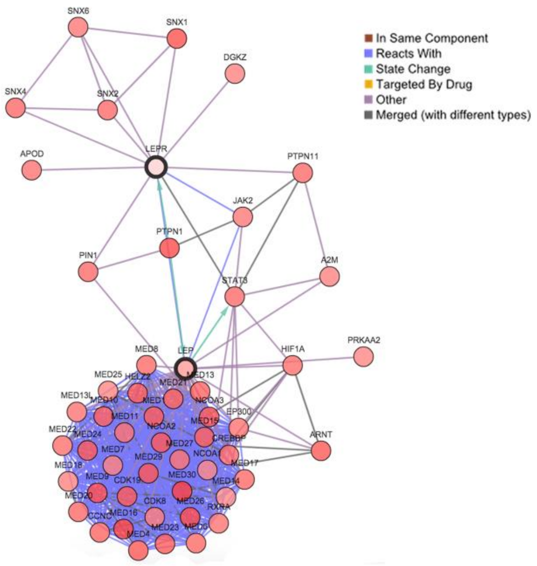

C)

Average abundance rate Z-score (phosphorylation)				
Protein	unaltered	altered	tendency	p-value
RB1	-0,07	0,18	↑	0,062
WWTR1	-0,05	0,17	↑	0,073
EGFR	-0,04	0,17	↑	0,09
YAP1	-0,03	0,17	↑	0,09
CHEK1	-0,03	0,17	↑	0,099

Average abundance rate Z-score (protein levels)				
Protein	unaltered	altered	tendency	p-value
MAP2K1	-0,07	0,29	↑	0,002
ERBB3	0,05	-0,21	↓	0,013
XBP1	0,05	-0,22	↓	0,014
INPP4B	0,05	-0,19	↓	0,038
ERBB2	0,03	-0,14	↓	0,044

Supplementary Figure 6: Relationship between the mRNA abundance of leptin and OB-Rb and DNA copy-number alterations (CNA), and the effect of high mRNA in the network neighborhood. A) *Upper panel*: a plot showing the relationship between leptin mRNA abundance and CNA in the leptin gene in serous ovarian cancer. *Lower panel*: a plot showing the relationship between OB-Rb mRNA abundance and CNA (DNA copy-number alteration) in the OB-Rb gene in serous ovarian cancer. Homdel, homozygously deleted; Hetloss, heterozygously deleted; Diploid, two alleles present; Gain, low-level gene amplification event; Amp, high-level gene amplification event. Blue dots indicate individual cancers. B) Graphical representation of the network neighborhood in high leptin/OB-Rb mRNA expressing serous ovarian cancers. C) Summary tables for the top five genes experiencing major changes in protein levels (*lower table*) and phosphorylation (*upper table*) in high leptin/OB-Rb-expressing serous ovarian cancers.
